# Description of the female of *Haematonotus jenisi* Krajcik, 2006 and placement of the species within the genus *Atrichiana* Distant, 1911 (Coleoptera, Scarabaeidae, Cetoniinae)

**DOI:** 10.3897/zookeys.561.6136

**Published:** 2016-02-08

**Authors:** Renzo Perissinotto

**Affiliations:** 1 DST/NRF Research Chair, Nelson Mandela Metropolitan University, Summerstand South Campus, P.O. Box 77000, Port Elizabeth 6031

**Keywords:** Scarabaeidae, Cetoniinae, genus *Atrichiana*, genus *Haematonotus*, revised status, female description, iSimangaliso Wetland Park, South Africa

## Abstract

The male of *Haematonotus jenisi* Krajcik, 2006 was described on the basis of a sole holotype specimen. A number of new specimens have recently been collected from the False Bay area of the iSimangaliso Wetland Park, in northeastern KwaZulu-Natal, including a number of females, thereby allowing the description of this sex for the first time. The new series also reveals that most morphological characters are actually typical for the genus *Atrichiana* Distant, 1911, rather than *Haematonotus* Kraatz, 1880. For this reason, it is here proposed that this species be placed within *Atrichiana*, thus becoming the second species of a genus that was previously regarded as monospecific, represented by the southern African endemic *Atrichiana placida* (Boheman, 1857). Like *Atrichiana placida*, *Atrichiana jenisi* exhibits a remarkable polymorphism, but also sexual dimorphism, particularly expressed at the level of the antennal clubs, which in the male are twice as long as in the female. Unfortunately, all specimens of the recent series were collected dead or drowning on the lake shores and therefore no further information on their life cycle and biology in general could be obtained. It seems that the species may represent a micro-endemism restricted to the Western Shores of Lake St Lucia, with habitat associated to Sand Forest and possibly also Northern Coastal Forest vegetation types.

## Introduction

Krajcik (2006) described *Haematonotus jenisi* on the basis of the single male holotype available at the time, providing a clear set of diagnostic characters and outstanding illustrations of the general habitus and genitalia of this interesting species. Its discovery was unexpected, as the area where it was collected (Dukuduku Forest, near Mtubatuba) falls within the iSimangaliso Wetland Park, a UNESCO World Heritage Site, where intensive surveys had been conducted in the past with the aim of understanding its full biodiversity richness. The Cetoniinae were among the key invertebrate groups investigated at the onset of the Park’s proclamation, within a dedicated project titled “Rare, threatened and endemic species project of the Greater St Lucia Wetland Park (Combrink and Kyle 2006). Krajcik’s surprise description of this new species triggered an escalation in research in the broader area of the Dukuduku section of the Park and further north, along the entire Western Shores of Lake St Lucia.

Understanding that the emergence of the adults of this species was most likely linked to major rainfall events, dedicated searches were conducted throughout the area during the summer season, immediately after a rain of > 20 mm. It was, however, only in April 2010 that two old dead specimens were found along the drying shores of False Bay, in the northern reaches of Lake St Lucia. It was not clear how long the specimens had been drowned in the lake, as the water at the time was hypersaline (salinity >100) and thus capable of preserving the tissues in relatively good state for a long time. After this, three other specimens were collected between November 2010 and November 2014, all in the same area of False Bay and drowned on the shores of the lake. The specimen collected in November 2013 was still alive, but died within a period of 12 hours. It was only at the beginning of February 2015 that 11 specimens in various states of decomposition were again retrieved from the lake shores of False Bay, in the wake of a rain of 55 mm which fell on January 15. This time, the series exhibited four females and also specimens with different colour patterns, thereby revealing a remarkable variability previously undetected and allowing the full description of the species.

Krajcik (2006) had placed the species within the genus *Haematonotus* Kraatz, 1880, but did not provide justification for this in the form of a set of typical diagnostic characters. Virtually all characters identified in this study clearly show that the species is actually much closer to *Atrichiana* Distant, 1911 than *Haematonotus* and, thus, it is here proposed that it be moved to the former genus, as its second species along with *Atrichiana placida* (Boheman, 1857).

## Methods

All specimens were collected drowned on the shores of False Bay, Lake St Lucia. Only one male specimen, collected in Nov 2013, was still alive while all other specimens were retrieved in various states of decomposition and immediately placed in ethyl acetate and later in a 10% formalin solution.

The description of morphological characters follows the terminology used by Krikken (1984) and Holm and Marais (1992). Specimen length was measured from the anterior margin of the clypeus to the apex of the pygidium. Specimen width represents the maximum width of the elytron. Photos of specimens dorsal and ventral habitus were taken with a Canon PowerShot G11 digital camera with macro setting. The background was removed from the photos using Microsoft Word 2010 (Picture Tools), in order to increase clarity of resolution. The Combine ZP Image Stacking Software by Alan Hadley (alan@micropics.org.uk) was used to obtain z-stacking composite images.

Repositories are abbreviated as follows: BMPC, Jonathan Ball and Andre Marais Private Collection, Cape Town, South Africa; DMSA, Durban Natural Science Museum, Durban, South Africa; RPPC, Renzo Perissinotto and Lynette Clennell Private Collection, Port Elizabeth, South Africa; TMSA, Ditsong National Museum of Natural History (formerly Transvaal Museum), Pretoria, South Africa.

## Taxonomy

### Atrichiana jenisi

Taxon classificationAnimaliaColeopteraScarabaeidae

(Kajcik, 2006) comb. n.

Figures 1 , 2

Haematonotus jenisi Krajcik, 2006: 15.

#### Material examined.

Sixteen male and four female specimens. 2 ♂: South Africa, KwaZulu-Natal, False Bay, Lister’s Point, 2 Apr 2010, found dead on lake shores, R. Perissinotto & L. Clennell leg. (RPPC); 2 ♂: same data as above but 3 Dec 2011 (DMSA, RPPC); 1 ♂: same data as above but Dukandlovu, 25 Nov 2013 found drowning on lake shores (RPPC); 7 ♂, 4 ♀: same data as above but 3 Feb 2015 (TMSA, BMPC, RPPC).

#### Genus placement.

The summary of characters listed here below and in Table 1 supports the placement of the species within *Atrichiana*, rather than *Haematonotus*. With the exception of the pronotal tubercle, which is a feature more typical of *Haematonotus*, although also visible in *Atrichiana* as “pronotal elevation” (Holm and Marais 1992), all other characters fit well into the range reported for the latter genus, including the male genitalia (see figure 8, p. 14 in Krajcik 2006). Neither *Atrichiana* nor *Haematonotus* are known to exhibit much longer antennal clubs in male than in female or a sharp hemi-pronotal carina. However, these are characters encountered in various genera and may not reflect generic synapomorphies, but rather adaptive convergence.

**Table 1.** Key generic characteristics of the genera *Haematonotus* Kraatz, 1880 and *Atrichiana* Distant, 1911, with closest matches in *Atrichiana jenisi* (Krajcik, 2006) highlighted in bold.

*Haematonotus* Kraatz, 1880	*Atrichiana* Distant, 1911
**Body velutinous, setose and with cretaceous areas on both dorsum and venter** (♂, except nigrito forms); or shiny, asetose and without cretaceous areas (♀)	Body dorsally velutinous and asetose; venter and pygidium shiny with cretaceous spots in both sexes
Clypeus strongly upturned apically	**Clypeus sharply upturned apically** (♂) ; **or mildly upturned and bilobate** (♀)
Pronotum trapezoid, with strong antescutellar arch **and prominent tubercle on anterior margin**	**Pronotum strongly attenuated forward, with base roundedly trisinuate** and anterior margin slightly protruding forward medially
Scutellum with round punctures denser in basal half and setiferous in ♂	**Scutellum flat and unsculptured**
Elytra with uneven costae raised and entire; with fine and dense sculpture	**Elytra with sutural costa roundedly raised, third and fifth less so; with diffuse sculpture**
Pygidium with basal and pre-apical lateral depressions; with bilateral cretaceous spots in ♂	**Pygidium with lateral depressions and cretaceous spots in both sexes (except darkest** ♀)
Mesometasternal process constrained and with protruding convexity	**Mesometasternal process laterally expanded and roundedly triangular**
Tarsi of average length	**Tarsi elongate, particularly in** ♂
Protibial denticle of ♂ well-developed, arched and produced forward	**Protibial denticle of** ♂ **extremely reduced and forwardly projected**
Meso- and metatibia with arched, uneven outer carina; weakly trilobed distally, with inner lobe more prominent	**Meso- and metatibia with no or poorly developed outer carina and spines**
Metatibial spur moderately ditated in ♀	**Metatibial spurs widely spatulate in** ♀
Aedeagus with ventral lobes of parameres distally slightly expanded, with small outer hooks and inconspicuous ventral setae	**Aedeagus with ventral lobes of parameres wider than dorsal lobes, with short setae ventro-apically**

#### Generic characters

**(Table 1)**:

1. Body dorsally velutinous with elytra asetose;
2. Extreme reduction and forward orientation of protibial denticles in male;
3. Tarsi generally elongate, particularly in male;
4. Presence of anterior elevation on pronotum (forming carina and tubercle – closer to *Haematonotus*);
5. Presence of white cretaceous spots on venter and pygidium;
6. Elytra with marked, uneven costae and diffuse sculpture;
7. Meso- and metatibia with no or poorly developed outer carina and spines, respectively;
8. Metatibial spurs widely spatulate in female;
9. Aedeagus with dorsal parameral lobes narrower than ventral and exhibiting short setae ventro-apically.

**Figure 1.**
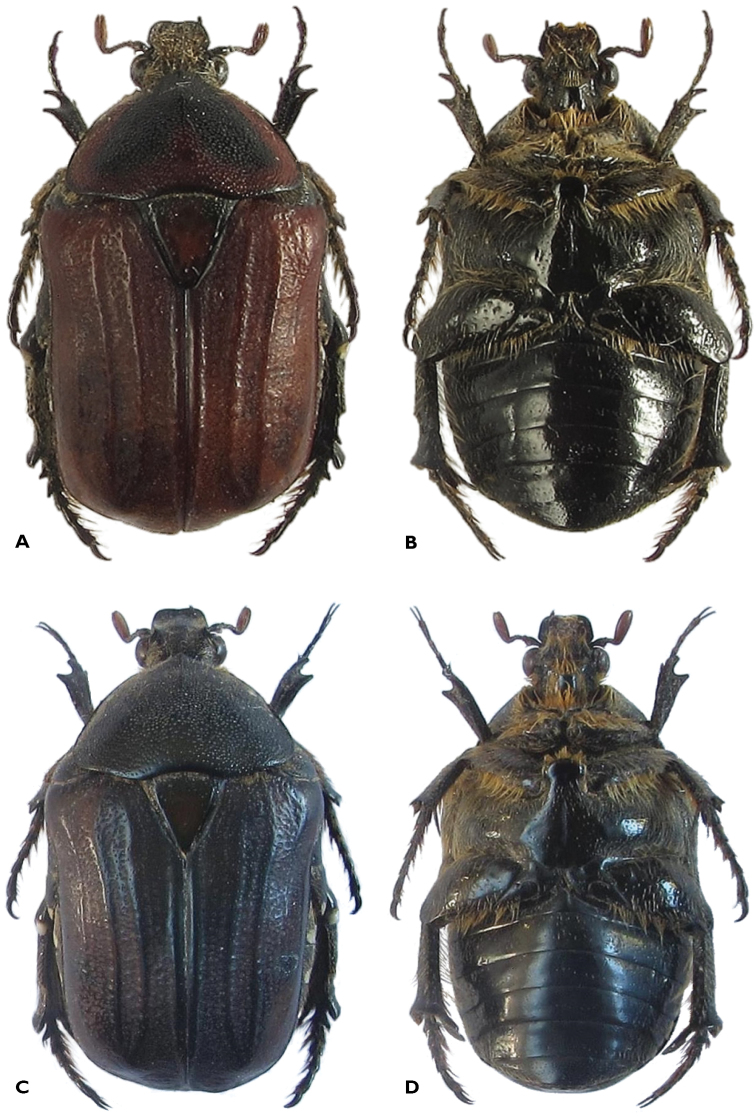
*Atrichiana jenisi*: **A** dorsal and **B** ventral habitus of light brown female specimen **C** dorsal and **D** ventral habitus of dark brown-green female specimen.

**Figure 2.**
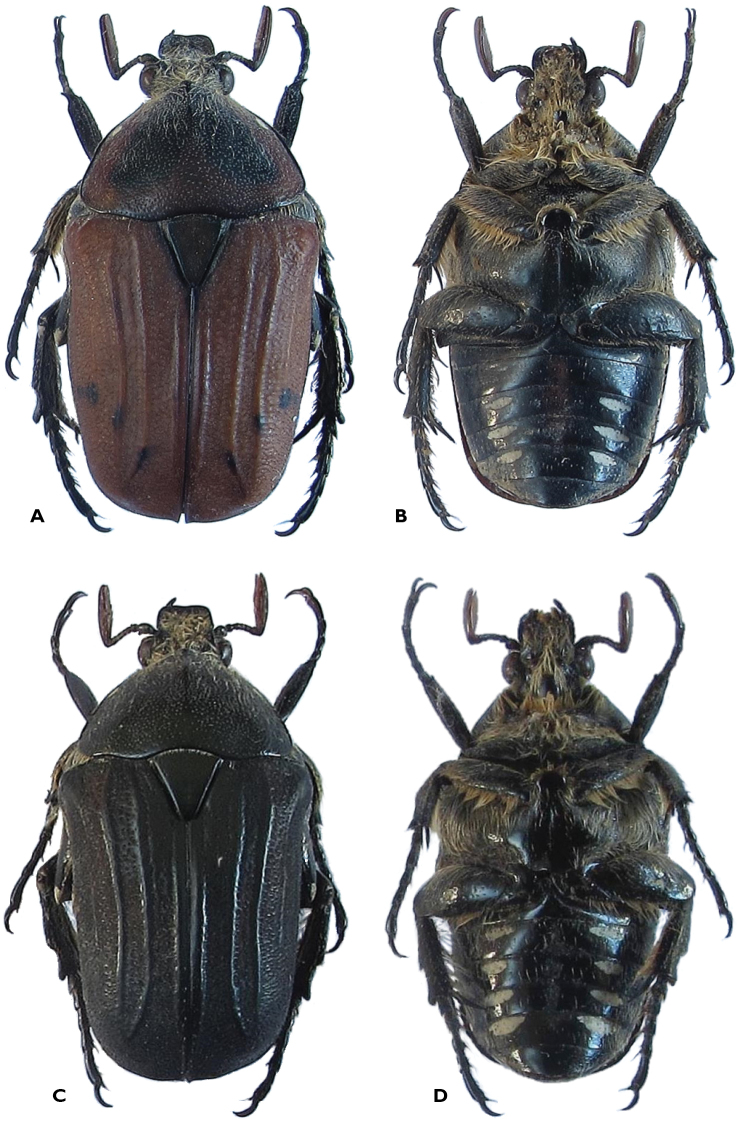
*Atrichiana jenisi*: **A** dorsal and **B** ventral habitus of light brown male specimen with black markings on apical half of elytra **C** dorsal and **D** ventral habitus of dark brown-green male specimen.

Species-specific characters (Figures 1A, C and 2A, C)

1. Antennal clubs twice as long in male compared to female;
2. Presence of cretaceous spot on each side of anterior pronotal declivity;
3. Presence of sharp vertical carina leading to tubercle on anterior half of pronotal midline;
4. Scutellum with narrow depression at middle of apical half.

#### Female description

(Figures 1). *Size*. Length 17.3–19.6 mm; width 10.5–11.2 mm (n = 4).

*Body*: Generally shorter and wider than male; background colour from black or dark green to dark brown with or without obvious maculae or bands (Figure 1A, C), alternatively light brown with two black/green bands on pronotum and three maculae on apical half of elytra (Figure 2A); presence of cretaceous spots on antero-lateral margins of pronotum, pygidium and base of metatibia.

*Head*. Black with scattered whitish, long setae and dense round punctures; clypeus slightly upturned, but far less than in male, with depression at center forming two lateral lobes, which together with marked lateral ridges enclose frons into teaspoon-like concavity; frons exhibiting smooth convex ridge at middle, reaching anterior pronotal margin.

*Pronotum*. Black, brown or dark green, matt with dense round punctures becoming progressively larger from the anterior and lateral margins to the center and posterior margin; anterior and lateral margins bearing thin, long but flattened creamy setae; exhibiting bilateral macula of white tomentum on lateral declivity, approximately at mid length; anterior margin as wide as eyes, but sharply raised with tubercle at center reaching disc as sharp carina; posterolateral angle smoothly rounded, posterior margin markedly sinuate anteriad of scutellum.

*Scutellum*. Dark green to brown or black; perfectly triangular, with apex slightly rounded; without or with few scattered punctures, mainly along lateral margins; with visible mid-depression on apical half.

*Elytron*. Exhibiting prominent sutural, discal and lateral costae, with latter two arching markedly towards apical declivity; sparse short creamy setae occurring only on lateral and apical declivities; diffuse crescent to horseshoe sculpture throughout surface, except on costal ridges; colour varying from almost completely black to dark green and even light brown, in latter case with three dark maculae on apical half of elytra.

*Pygidium*. Dark green to black or brown, with bilateral cretaceous spots drastically reduced or absent; exhibiting short creamy setae near sides and apex; with fine, wrinkled sculpture throughout surface.

*Appendages*. Antennal clubs reddish brown, of normal length, shorter than pedicel and flagellum combined; pedicel and flagellum with scattered but long, erected creamy setae; protibia broadly expanded, with short and scattered creamy setae, bidentate with apical tooth hypertrophic and falciform; meso- and metatibia with long, dense creamy setae, with no or poorly developed outer carina and spines, respectively; metatibial spurs widely spatulate; profemur, mesepimeron, mesofemur and metafemur all covered with very long creamy setae.

*Underside*. Shiny black to dark green; creamy, long setae throughout surface, except at center of metasternum and abdominal sternites; mesosternal lobe flat and roundedly expanded; abdominal sternites with visible convexity at center and without any white tomentose markings between midline and lateral margins (Figure 1B, D).

*Remarks. Atrichiana jenisi* shows a marked sexual dimorphism, with the female exhibiting antennal clubs about half the length of that of its male counterpart and a shorter clypeus, with far less upturned margins than in the male (Figures 1 and 2). The female also has a shorter and broader body shape than the male and does not exhibit large white tomentose markings either on the pygidium or the abdominal sternites. Its protibial surface is far broader than in the male and its apical teeth are strongly developed, indicating an extraordinary adaptation to fossorial activity. It does, however, possess fully developed and functional wings, which are not significantly different to those observed in the male. *Atrichiana jenisi* is closely related to the only other species currently known within the genus, *Atrichiana placida*. It differs from the latter in having a generally more oblong body, more marked elytral costae and a predominatly dark green to black, rather than light brown background colour. The most diagnostic characters separating the two species may, however, lie in *Atrichiana jenisi* exhibiting a carina in the mid anterior third of its pronotal disc, a bilateral white macula on the lateral declivity of the pronotum and a vertical depression in the apical half of its scutellum (Figures 1A, C and 2A, C). None of these characters are present in *Atrichiana placida*. Otherwise, the two species share a remarkable variability in background colouration, from black to dark or light brown, with various shades of dark maculation. A dark green ground colour is not yet known in *Atrichiana placida*, while this is predominant in *Atrichiana jenisi*. The two species are sympatric on the Western Shores of Lake St Lucia, where *Atrichiana placida* appears to be marginally present in low abundance while showing its preference for the Eastern Shores of the lake and the coastal dune area of the southern Indian Ocean. Conversely, *Atrichiana jenisi* is completely absent from the coastal area proper and appears to be restricted to the more inland forests, from Dukuduku in the south to False Bay in the north. No records beyond this narrow area are currently known for the species and it is therefore likely that it may be a micro-endemic of the Western Shores of Lake St Lucia.

## Discussion

It is remarkable that *Atrichiana jenisi* was discovered only very recently, despite the intensive surveys that have been undertaken historically in the area of the iSimangaliso Park (Combrink and Kyle 2006), with its status of UNESCO World Heritage Site. This confirms the extraordinary value of this park, as hotspot of global biodiversity and center of endemism.

**Figure 3.**
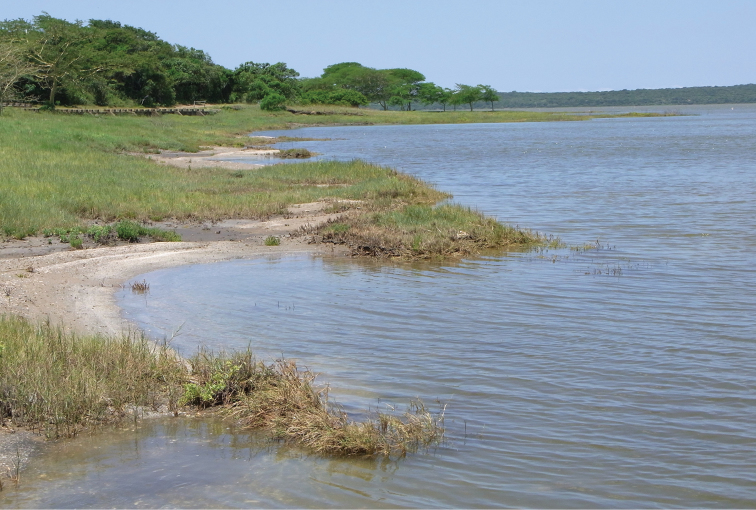
Shoreline of False Bay near Lister’s Point, where the first drowned specimens of *Atrichiana jenisi* were collected (Photo: Lynette Clennell, 22 Dec 2012).

**Figure 4.**
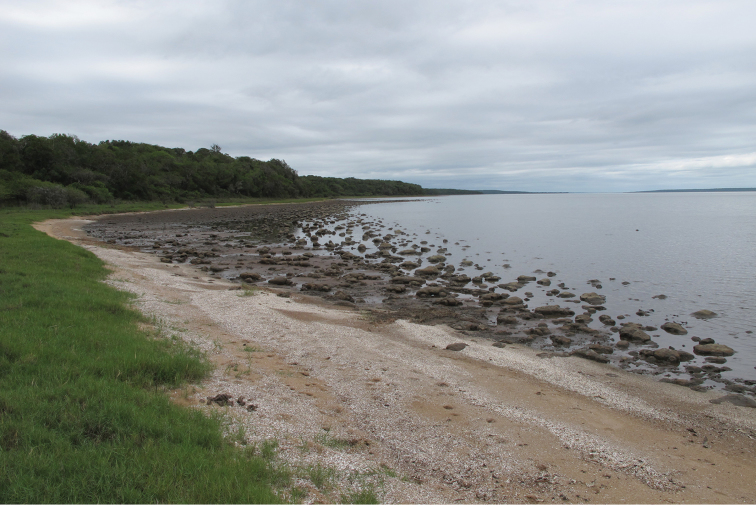
Dukandlovu shoreline, southern False Bay, where several specimens of *Atrichiana jenisi* were retrieved dead in January/February 2015 (Photo: Lynette Clennell, 1 February 2015).

Virtually nothing is yet known about the biology of the species, as all specimens known so far have been retrieved dead or drowning on the shores of False Bay in Lake St Lucia (Figures 3 and 4). An exception is provided by the holotype described by Krajcik (2006), which was reportedly collected between Mtubatuba and Dukuduku in December 2007 (cf. specimen data label). This is most likely in the area of the old picnic site on the road to the St Lucia Estuary, which has now been enclosed within the game fence of the iSimangaliso Wetland Park and rehabilitated into the wilderness of the Dukuduku Forest section. At the time of the holotype collection, the picnic site represented the only resting point along the road, with access to the indigenous forest habitat.

Adults are most likely unable to feed, as no specimens have been collected either in fruit baited traps, on flowers or sap flows, despite the intense collections that were undertaken in the past 15 years, during the period leading to the proclamation of the Park as a World Heritage Site and afterwards (Combrink and Kyle 2006). What is even more extraordinary is that no specimens have so far been observed active in flight or on the ground during this period, apart from one male specimen collected in November 2013, which had fallen into the water at False Bay and was climbing up the stem of a sedge clump. All other specimens found in Lake St Lucia were already dead for some time, but most were still in a good state of preservation due to the high salinity of the lake waters at the time.

It appears that adults are active for a relatively short period of time at the onset of the rainy season, from November to February. Adult emergence is obviously linked to major rainfall events, with all fresh specimens collected immediately or shortly after substantial rain (> 20 mm). For instance, most specimens known so far were collected along the shores of False Bay on the 3^rd^ of February 2015 (Figures 3 and 4). They had all died a while before, as tissue decomposition was already well underway, despite the high salinity of the water (around 30). The summer of 2014–2015 had been particularly dry in the region and the first substantial rain in the False Bay area fell only between the middle and the end of January (69 mm during 15–17 Jan 2015, 54 mm during 28–30 Jan 2015; False Bay Park Meteo Station). As neither adult activity nor freshly dead specimens were observed during the survey undertaken between January 31 and February 3, it seems most likely that all the dead specimens had emerged with the first rain event of 15–17 January.

*Atrichiana jenisi* appears to be an endemic of the Sand Forest, possibly extending into the Northern Coastal Forest vegetation unit (types FOz 8 and FOz 7, respectively, of Mucina and Rutherford 2006). Both vegetation units are seriously threatened in the Maputaland bioregion, with the Sand Forest actually regarded as critically endangered because of its vulnerability and the socio-economic pressure currently exerted on the region (Mucina and Rutherford 2006). However, the specific habitat of the larval stages of *Atrichiana jenisi* remains unknown. This, despite the many Cetoniinae collections and rearing of larvae that have taken place in the area over a relatively long period of time. It seems likely that its habitat may be either patchy and extremely restricted or otherwise unusual (e.g. aerial epiphytic deposits, bird nests etc.). Given the importance of this area and its unique biodiversity to the overall conservation and sustainability of the iSimangaliso Wetland Park, further investigations are required in order to unravel the enigmatic life cycle and ecology of this endemic species.

## Supplementary Material

XML Treatment for Atrichiana jenisi
